# Editorial: The Stretch-Shortening Cycle of Active Muscle and Muscle-Tendon Complex: What, Why and How It Increases Muscle Performance?

**DOI:** 10.3389/fphys.2021.693141

**Published:** 2021-05-20

**Authors:** Wolfgang Seiberl, Daniel Hahn, Geoffrey A. Power, Jared R. Fletcher, Tobias Siebert

**Affiliations:** ^1^Human Movement Science, Department of Human Sciences, Bundeswehr University Munich, Neubiberg, Germany; ^2^Human Movement Science, Faculty of Sport Science, Ruhr University Bochum, Bochum, Germany; ^3^School of Human Movement and Nutrition Sciences, University of Queensland, Brisbane, QLD, Australia; ^4^Neuromechanical Performance Research Laboratory, Department of Human Health and Nutritional Sciences, College of Biological Sciences, University of Guelph, Guelph, ON, Canada; ^5^Department of Health and Physical Education, Mount Royal University, Calgary, AB, Canada; ^6^Department of Motion and Exercise Science, University of Stuttgart, Stuttgart, Germany; ^7^Center for Simulation Science, University of Stuttgart, Stuttgart, Germany

**Keywords:** eccentric contraction, passive elastic energy, stretch-reflex, performance enhancement, history dependence, plyometric exercise

## Introduction

**Figure 1:**
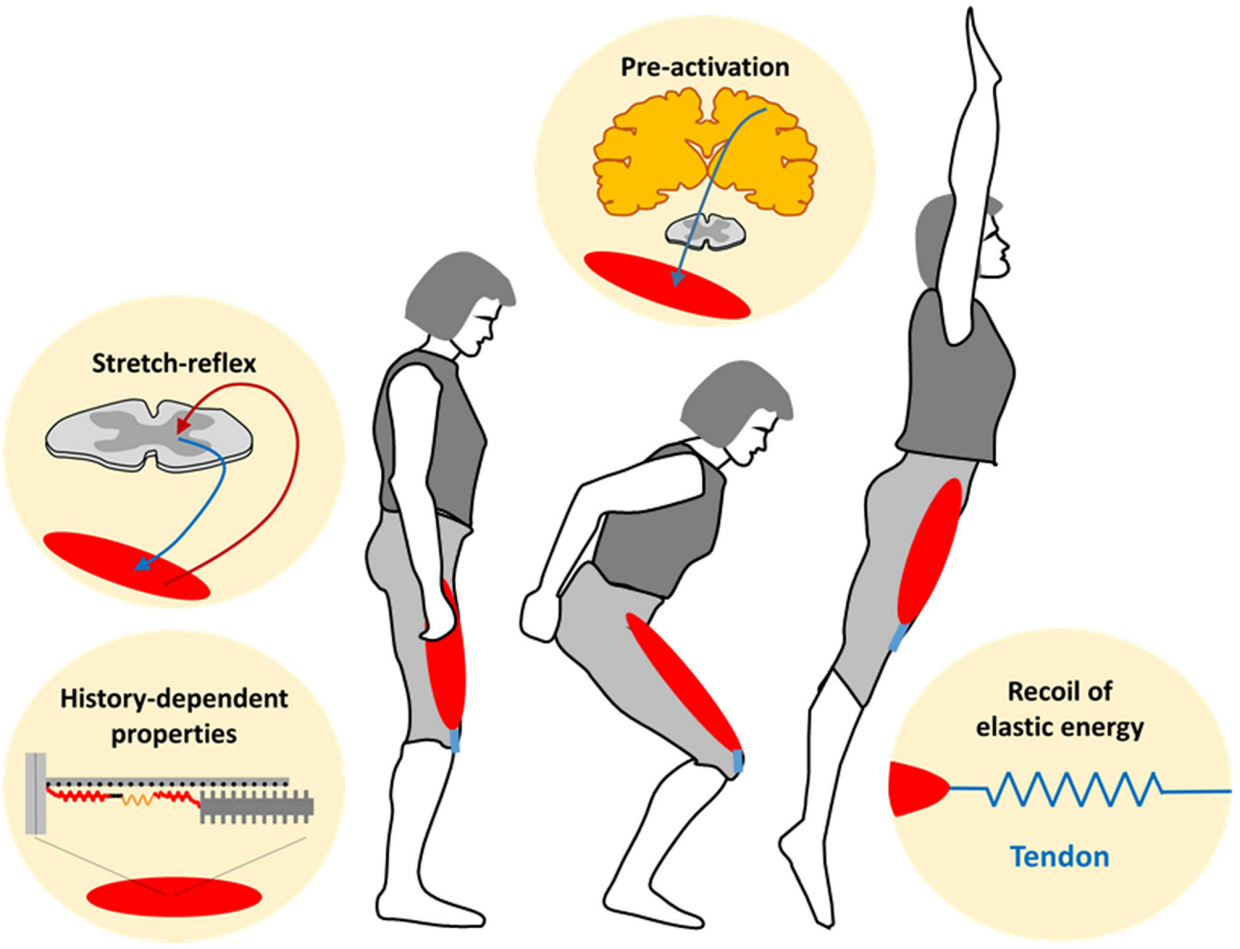
Potential mechanisms contributing to the performance enhancing effect of *in vivo* muscle-tendon unit stretch-shortening cycles.

During a stretch-shortening cycle (SSC), a muscle is first actively stretched before it actively shortens (Cavanagh and Komi, 1979). Intriguingly, the force, work, and power output during the shortening phase of a SSC is enhanced compared with shortening that is not preceded by active stretch (Cavagna et al., 1965). Since then, the SSC-effect has fascinated researchers and several mechanisms underlying the SSC-effect have been considered. These mechanisms include neuromuscular pre-activation, stretch-reflex contributions, and recoil of elastic energy stored in tendons (van Schenau et al., 1997). Furthermore, it was suggested that following the initial stretch, the force production is enhanced at the sarcomeric level during shortening of SSCs (Cavagna et al., 1968). While this force enhanced mechanism hasn't received much acceptance, it was recently revisited and linked to the plastic history-dependent properties of muscle (Seiberl et al., 2015). Since then, a new series of SSC studies focused on the history-dependent properties of stretch-induced force enhancement provided strong support for their relevance in SSCs. Therefore, the aim of this Research Topic was to reignite a holistic debate on the mechanisms contributing to the SSC-effect (Figure 1), as well as on the relevance of SSCs for movement and training.

## Contractile Mechanisms Underlying SSC

Four papers in this Research Topic focused on the underlying contractile mechanisms that might contribute to the SSC-effect. Tomalka et al. (a) used skinned single fiber preparations to show that part of the SSC-effect is sarcomere based. By using a cross-bridge inhibitor, their data indicates that a non-cross bridge viscoelastic element contributes to the SSC-effect. This suggestion was further supported by Hessel et al. who, in their study, also used skinned muscle fiber preparations and a myosin inhibitor, as well as a whole muscle preparation from *mdm* mice, which had a small deletion of titin's N2A region. Based on the combined results from both papers, the giant spring-like protein titin appears to contribute to the SSC-effect. In their second study, Tomalka et al. (b) showed that the SSC-effect in skinned fibers increases with SSC velocity. This again points toward a contribution of non-cross-bridge structures to the SSC-effect, which might be explained by the viscoelastic properties of the structural protein titin. The fourth study that used skinned fibers by Joumaa et al. investigated the energy cost of force production following SSCs and following shortening contractions of identical speed and magnitude. While their data support the contribution of history-dependent muscle properties to the SSC-effect (Seiberl et al., 2015), energy cost per unit of force was not different during an isometric steady-state following SSCs compared with shortening without preceding stretch. Joumaa et al. suggest that the increase in total force observed following SSCs was achieved with an increase in the proportion of attached cross-bridges and titin stiffness. As their data mainly refer to the steady-state following SSCs, no conclusion can yet be drawn regarding the metabolic cost *during* SSCs.

## *In Vivo* Human SSC

Another four studies in this Research Topic focused on SSCs of *in vivo* human muscles. Similar to the *in vitro* studies described above, Groeber et al. investigated whether the history-dependent properties of muscle contribute to the SSC-effect in the human quadriceps femoris. For electrically and voluntarily activated contractions at activation levels ranging from 20 to 100%, they observed a consistent SSC-effect. Residual force depression following SSCs did not differ or was less compared with shortening contractions without preceding stretch. As residual force depression is known to increase with the work produced during shortening (Chen et al., 2019), their finding provides indirect support for the contribution of history-dependent muscle properties to the SSC-effect. Similarly, but in a more applied setting, Held et al. found a SSC-effect in terms of a 10% higher power output during rowing, when rowing was performed with a countermovement before leg extension compared with a leg extension without such countermovement. However, in their study, the question remains whether a SSC occurred in the muscle, the tendon or both. This question was investigated by Aeles and Vanwanseele for the medial gastrocnemius during jumping. In their study, by using B-mode ultrasound, they found that for this specific movement a SSC did occur at the level of the muscle-tendon unit and the tendon but not the muscle fascicles. Accordingly, related to where the SSC occurs, different mechanisms are likely at play. To better understand SSCs of *in vivo* human muscles, Wearing et al. performed a proof of concept regarding the reliability of transmission mode ultrasound in quantifying the instantaneous modulus of elasticity of human Achilles tendon during repetitive submaximal hopping. Their results showed that the technique allows to reliably monitor ultrasound velocity and to detect changes in the instantaneous elastic modulus of the tendon, which may provide further insights into *in vivo* SSC performances.

## Short- and Longterm Adaptations to SSC

Finally, four articles of this Research Topic dealt with short- and long-term adaptation of the muscle-tendon unit to SSCs and whether SSCs are a suitable training tool, typically referred to as plyometric training. Kositsky et al., demonstrated acute main effects after exhaustive SSCs: decreased voluntary strength and joint stiffness, and increased resting fascicle lengths of medial gastrocnemius muscle. On a larger timescale, Hammami et al. showed that a 10 weeks upper and lower limb plyometric training program improved many measures of physical performance in young female handball players. Similarly, after a 6-week plyometric training intervention, Ruffieux et al. found significant increases in jump performance. More specifically, they concluded that (at least for non-professional female volleyball players) training with a high percentage of slower SSC jumps, such as counter-movement jumps, is more effective than a training with a high percentage of fast SSCs, such as drop-jumps. Therefore, slower SSCs during CMJs seem to be more specific for these players and tasks. To better understand the time-course of changes in muscle function and morphological parameters in response to SSCs, Monti et al. compared muscle torque and power combined with muscle architectural changes after 2, 4, and 6 weeks of plyometric training. They showed rapid increases in muscle volume, fascicle length, pennation angle, torque, and power in healthy younger adults following the training. They also pointed out that SSC exercise is likely beneficial for neuronal adaptations to performance, as not all enhancements in muscle power were explainable by increases in cross-sectional area or muscle volume.

## Limitations and Future Perspectives

The main limitations of the studies covered in this Research Topic are that (1) skinned muscle fiber experiments provide insight into potential contractile mechanisms but that they do not necessarily represent muscle function under physiological conditions. Conversely, (2) the *in vivo* studies represent physiological muscle function but suffer from the problem of identifying underlying mechanisms. A major limitation of (3) the training studies is that the mechanisms that trigger specific adaptations remain unclear and that it is very difficult to match different training regimes to make them comparable. Especially for *in vivo* human muscle research, this Research Topic showed that it is crucial for future studies to distinguish between the behavior of the muscle-tendon unit, the tendon, and the muscle fascicles. Further, although a broad field of SSC research is covered in this Research Topic, the interplay of potential mechanisms is not yet well-understood, and research on neuromuscular factors and modeling approaches are missing in this collection. Accordingly, SSCs remain a topic of high relevance and great scientific interest.

## Author Contributions

All authors contributed to the manuscript and approved the final version.

## Conflict of Interest

The authors declare that the research was conducted in the absence of any commercial or financial relationships that could be construed as a potential conflict of interest.
